# Correction: Sutcliffe et al. Common Oral Medications Lead to Prophage Induction in Bacterial Isolates from the Human Gut. *Viruses* 2021, *13*, 455

**DOI:** 10.3390/v15010025

**Published:** 2022-12-21

**Authors:** Steven G. Sutcliffe, Michael Shamash, Alexander P. Hynes, Corinne F. Maurice

**Affiliations:** 1 Department of Microbiology and Immunology, McGill University, Montreal, QC H3A2B4, Canada; 2 Department of Medicine, McMaster University, Hamilton, ON L8S4L8, Canada

## Error in Figure

In the original publication [1], there were two mistakes in Figure 4B as published. The first error is a duplication of the Mitomycin C and Norfloxacin panels, which took place during the figure layout process in Power Point. The published Mitomycin C column is the correct one, whereas the published Norfloxacin column was a duplicated version of Mitomycin C. The second mistake is also a duplication concerning the Normalized read coverage profiles for prophage 2 in the Mitomycin C, Norfloxacin, and Ampicillin treatments. In this case, the Ampicillin plot is correct, but the Mitomycin C and Norfloxacin are duplications of the Ampicillin treatment. This occurred because of a typo in the R code used to export figures. The corrected Figure 4B appears below.


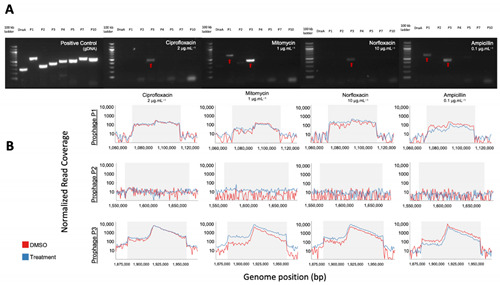


In the original publication, there was also a mistake in Supplementary Figure S3 as published. We inverted the water and ciprofloxacin labels by mistake for all bacterial growth curves, except for *B. caccae*, which was correct. The authors state that the scientific conclusions are unaffected. This correction was approved by the Academic Editor. The original publication has also been updated.
