# Supplementary material for: Common Oral Medications Lead to Prophage Induction in Bacterial Isolates from the Human Gut
Source: Viruses. 2021 Mar 11;13(3):455. doi: 10.3390/v13030455 (PMC8000485; doi:10.3390/v13030455)
Supplement: Supplementary file 1 [file viruses-13-00455-s001.pdf]

Supplementary Material:

Common oral medications lead to prophage induction in bacterial isolates from the human gut

Supplementary Figures

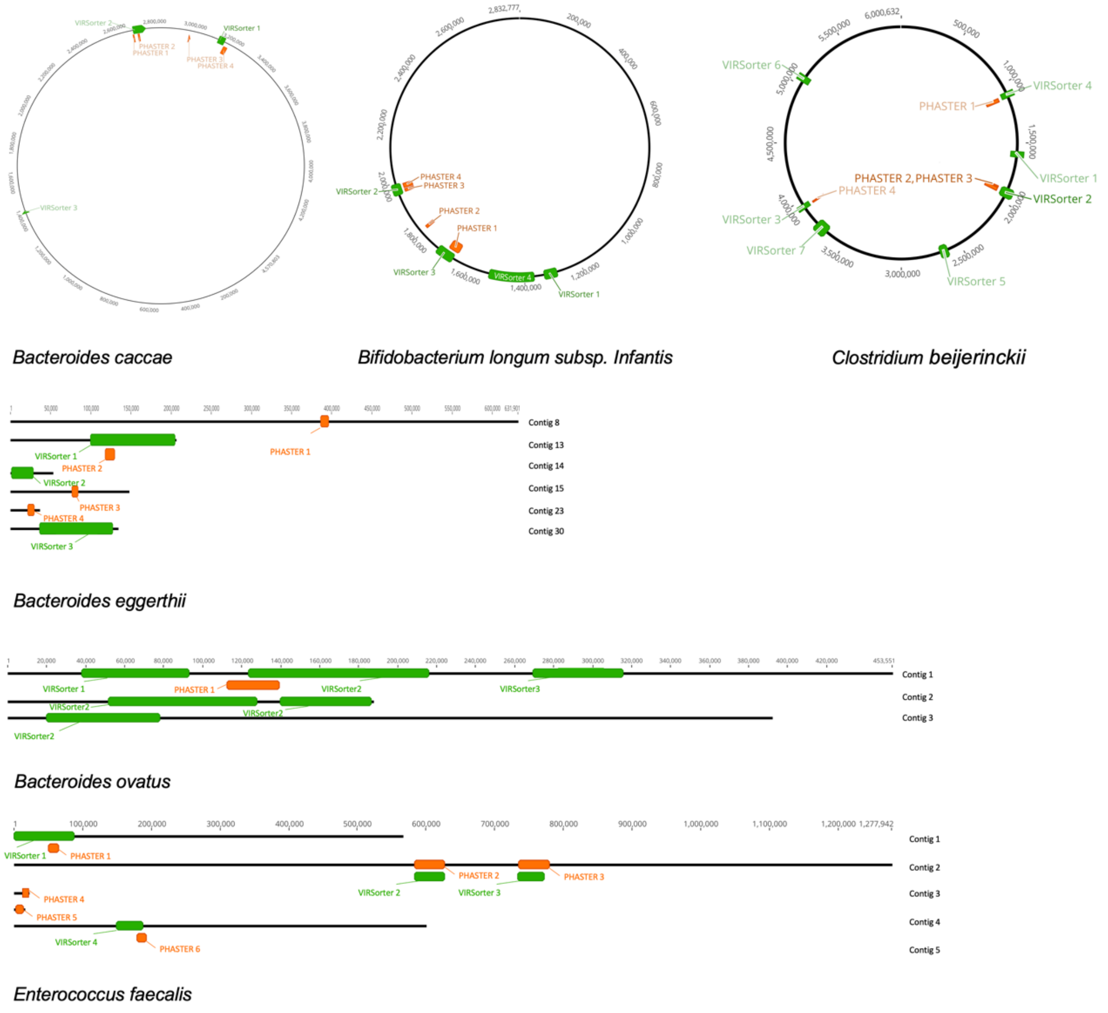

Supplementary Figure S1. Location of putative prophages within bacterial chromosome or contig: Prophages were detected by PHASTER Web Server (Default settings) and VirSorter. VirSorter was run with the default options except for the following: use viromes reference database (this database includes sequences from viral RefSeq as well as those obtained from aquatic and human gut, lung, and saliva environments) (--db 2), use DIAMOND for protein

alignment (--diamond). For contiguous genomes *(B. eggerthii*, *B. ovatus*, and *E. faecalis*) only contigs containing prophages are shown (Made in Geneious 2020 0.05).

A

Primer	Sequence (5'➡3')	Description
Clostridium beijerinckii (ATCC 51743)		
**C.beij-P1-1F**	TTGGCGATACACCACAAGAG	portal protein (CBEI_RS04885)
**C.beij-P1-1R**	TCTTCACCTCCTCCCAAGTC	
**C.beij-P2-1F**	ATCCTGTTTTTGCTGACGATGG	portal protein (CBEI_RS07205)
**C.beij-P2-1R**	AGCACCTTGGAATGGTTGTCC	
**C.beij-P3-1F**	CGGTTAAAGCTAATTGGGGAC	tail sheath protein (CBEI_RS08600)
**C.beij-P3-1R**	ATTGTTCCTGTGGCTCCTGA	
**C.beij-P4-1F**	TTAGATAAATGCCAGGGCTGC	RNA polymerase sigma factor (CBEI_RS11590)
**C.beij-P4-1R**	TGTTGCCTACGCTACCAATG	
**C.beij-P5-1F**	TATGGCGATGGTCCTGACAC	hypothetical protein (CBEI_RS13335)
**C.beij-P5-1R**	ATCTCTCGCATGGGTCTTCC	
**C.beij-P7-1F**	TGCCTTTCCACTTCCTTCACC	baseplate assembly protein (CBEI_RS17380)
**C.beij-P7-1R**	GAAGCTCCAGAGGTGCCCAA	
**C.beij-P10-1F**	AGCCTGTCCCATCTTGTTGAG	putative tail protein/transcrp. regulator (CBEI_RS22455)
**C.beij-P10-1R**	ACAATGAAACCAAGGGTGCC	
**C.beij-DnaA-1F**	GCTGCGAACCTCTGTCTATTTC	DnaA gene, chromosomal DNA replication initiation factor
**C.beij-DnaA-1R**	TGGTGATTCTGCAACTGCCA	

B

			**Normalized Coverage**		
**Xenobiotic**	**Dose**	**Prophage**	**Vehicle Control**	**Treatment**	**Fold Change**
Clostridium beijerinckii (ATCC 51743)					
**Ampicillin**	0.1 μg/mL	P1	111.399	53.556	0.481
		P3	1144.519	649.626	0.568
**Mitomycin C**	1 μg/mL	P1	77.860	74.031	0.951
		P3	716.779	1691.936	2.360
**Norfloxacin**	10 μg/mL	P1	157.537	246.761	1.566
		P3	839.069	1544.530	1.841
**Ciprofloxacin**	2 μg/mL	P1	152.458	162.869	1.068
		P3	1307.908	1436.539	1.098

Supplementary Figure S2. PCR Identified Prophages of *C. beijerinckii:* A) Primer sequence for each prophage region and description of protein associated with sequence. B) Normalized

read coverage by each prophage region after induction treatment of shotgun sequenced vDNA.

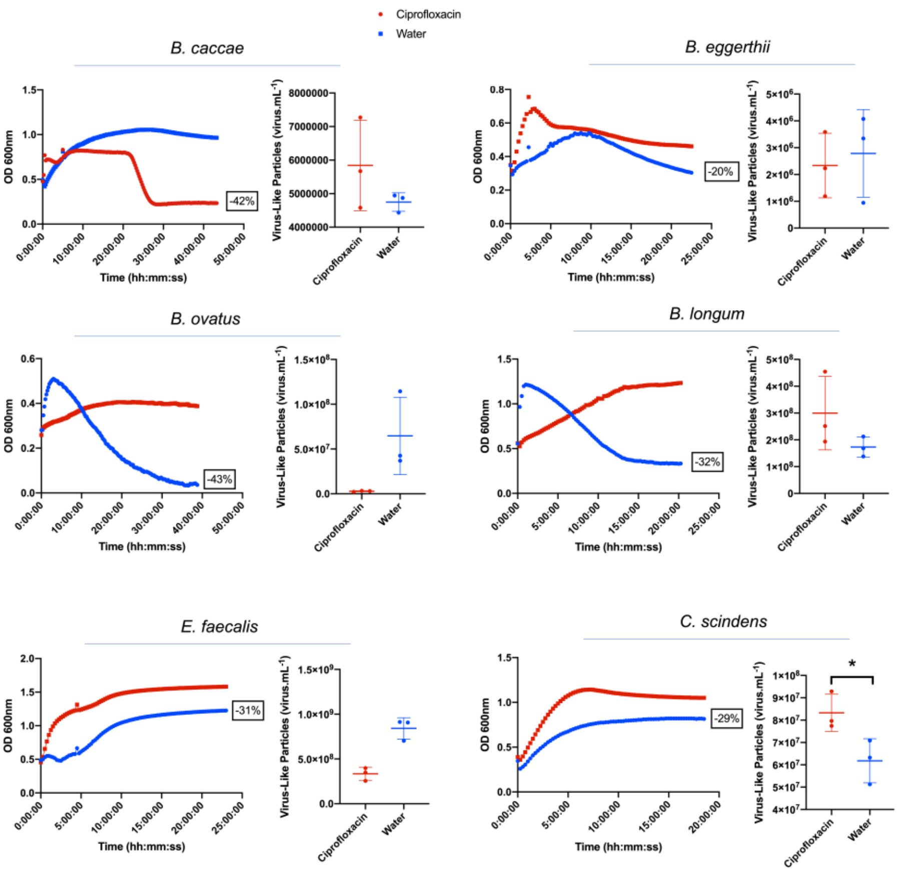

Supplementary Figure S3. Bacteria not inhibited by low dose of ciprofloxacin grown with higher concentrations (20µg.mL^{-1}) of ciprofloxacin dissolved in water (pH 6.5) as vehicle. Growth curves are mean OD 600nm values. Virus-like-particle plots show individual values (n=3), with mean (horizontal bar) and standard deviations (vertical bars). * represents $p < 0.05$ (unpaired t-test) between control and treatment.

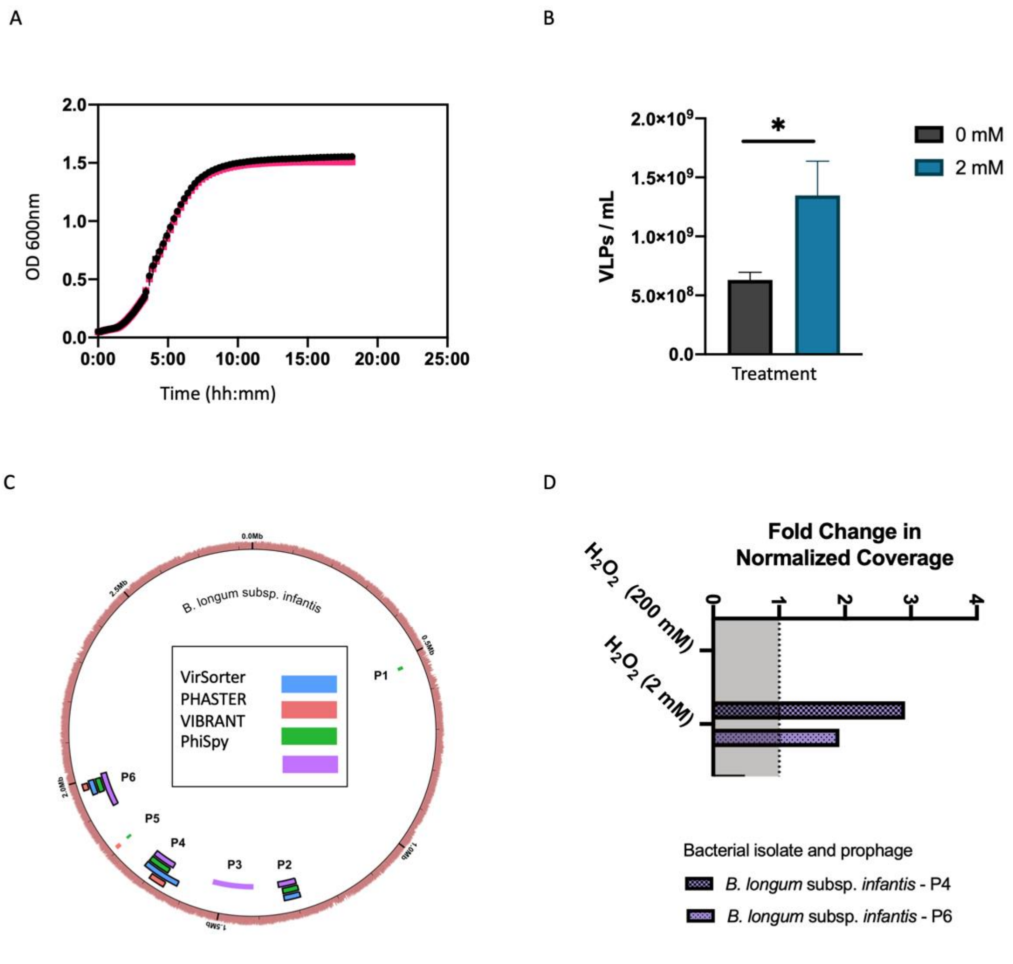

Supplementary Figure S4. Induction of *B. longum* prophage by hydrogen peroxide in absence of bacterial growth inhibition: A) Growth curve of *B. longum* in Control (Black) compared to exposure to 2mM hydrogen peroxide treatment showing no change in growth curve compared to (red) water control. B) Increase in VLPs after exposure to 2mM hydrogen peroxide compared to control (*, $p<0.05$) C) Detection of predicted prophage regions using VirSorter, PHASTER, VIBRANT, and PhiSpy D) Fold change in read coverage compared to control of vDNA obtained by shotgun sequencing after hydrogen peroxide treatment for prophage regions P4 and P6 .

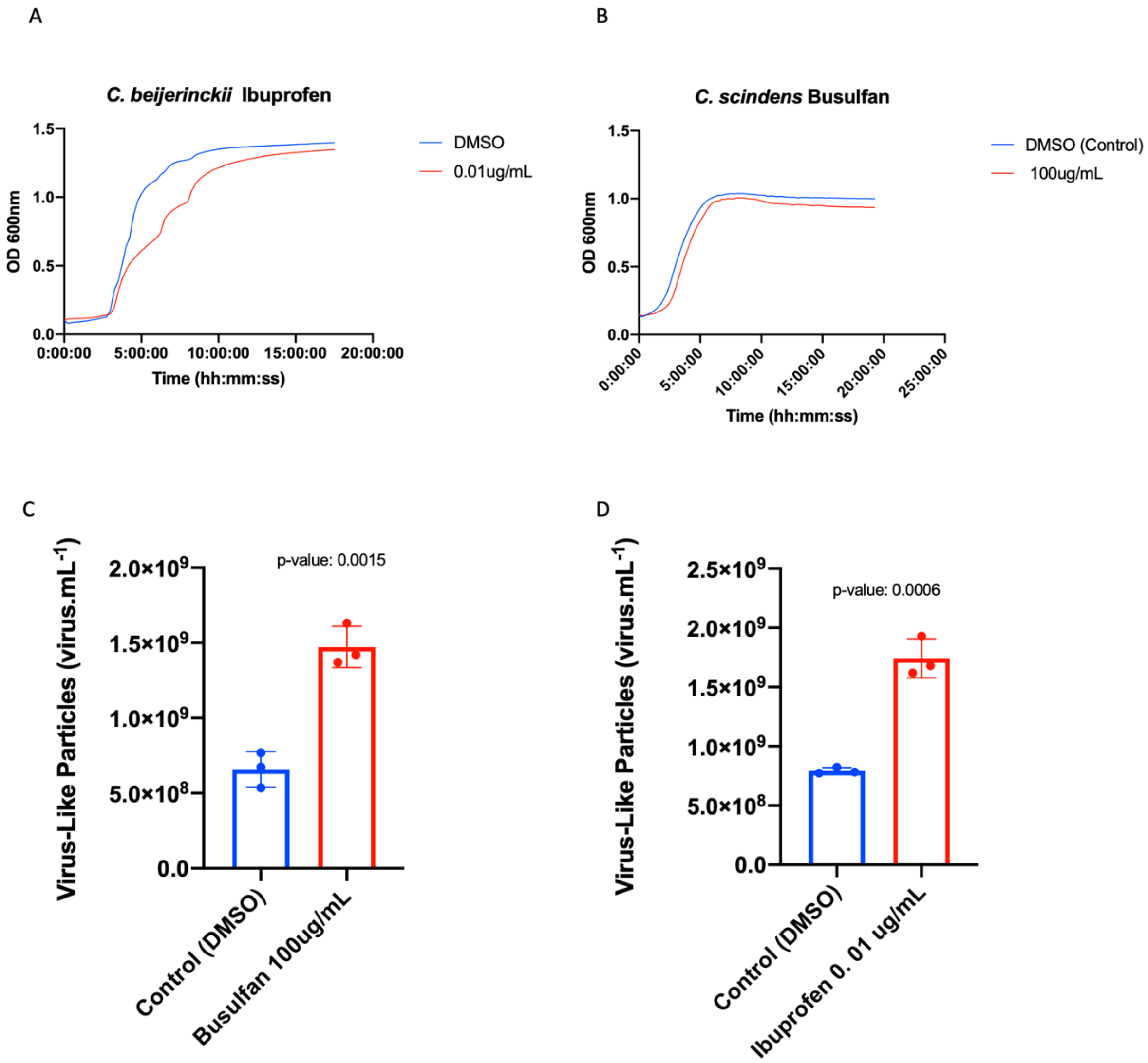

Supplementary Figure S5: Prophage Induction without antibacterial activity of *C. beijerinckii* and *C. scindens:* A) Growth curve of *C. beijerinckii* with Ibuprofen and the corresponding B) significant increase in VLPs as well as C) *C. scindens* with Busulfan and corresponding D) significant VLP increase. (Significance calculated by unpaired two-tailed t-test)

Supplementary Tables

Drug	Minimum Range of Dose (mg)	Maximum Range of Dose (mg)	Minimum Range of Bioavailability (%)	Maximum Range of Bioavailability (%)	Minimum Amount in Colon (µg.mL-1)	Maximum Amount in Colon (µg.mL-1)
**Ampicillin**	250	4000	50	90	44.56	3565.06
**Ciprofloxacin**	100	1000	30	40	106.95	1247.77
**Norfloxacin**	400	800	30	40	427.81	998.22
**Streptonigrin**	0.2	0.2	48	72	0.10	0.19
**Mitomycin C***	-	-	-	-	-	-
**Diclofenac**	50	75	50	50	44.56	66.84
**Ibuprofen**	300	1200	80	80	106.95	427.81
**Tolmetin**	200	700	50	90	35.65	624.00
**Busulfan**	2000	2000	70	70	1069.52	1069.52
**Fludarabine**	10	10	58	58	7.49	7.49
**Acetaminophen**	650	650	73	100	0.00	312.83
**Digoxin**	0.125	0.25	70	70	0.07	0.13

Supplementary Table S1. Maximum and minimum oral dose concentrations were calculated using oral doses[1] (Drug@FDA[2], ATC/DDD).[3] Bioavailability data[1] (t[4], q[5], and j[6]) was used as an approximation for how much of the drug is absorbed into the blood before entering the colon. The minimum and maximum estimated concentration were calculated based on the remaining dose in the estimated volume of the colon (561mL).[7]

Supplementary References

1. Goodman, L. S.; Brunton, L. L.; Chabner, B.; Knollmann, B. C., Goodman & Gilman's pharmacological basis of therapeutics. In 12th ed.; McGraw-Hill,: New York, 2011.
2. Administration, U. S. F. a. D. Drug@FDA www.fda.gov/drugsatfda (Dec 6),
3. Organization, W. H. ATC classification index with DDDs 2019. https://www.whocc.no/atc_ddd_index/ (Dec 6),
4. Stein, G. E., Review of the bioavailability and pharmacokinetics of oral norfloxacin. *Am. J. Med.* **1987,** *82*, (6B), 18-21.
5. Grindel, J. M., The pharmacokinetic and metabolic profile of the antiinflammatory agent tolmetin in laboratory animals and man. *Drug Metab. Rev.* **1981,** *12*, (2), 363-77.
6. Yin, W.; Karyagina, E. V.; Lundberg, A. S.; Greenblatt, D. J.; Lister-James, J., Pharmacokinetics, bioavailability and effects on electrocardiographic parameters of oral fludarabine phosphate. *Biopharm Drug Dispos* **2010,** *31*, (1), 72-81.
7. Pritchard, S. E.; Marciani, L.; Garsed, K. C.; Hoad, C. L.; Thongborisute, W.; Roberts, E.; Gowland, P. A.; Spiller, R. C., Fasting and postprandial volumes of the undisturbed colon: normal values and changes in diarrhea-predominant irritable bowel syndrome measured using serial MRI. *Neurogastroenterol Motil* **2014,** *26*, (1), 124-30.
